# Impact of Sodium Silicate Supplemented, IR-Treated Panax Ginseng on Extraction Optimization for Enhanced Anti-Tyrosinase and Antioxidant Activity: A Response Surface Methodology (RSM) Approach

**DOI:** 10.3390/antiox13010054

**Published:** 2023-12-28

**Authors:** Seda Nur Kabadayı, Nooruddin Bin Sadiq, Muhammad Hamayun, Nam-Il Park, Ho-Youn Kim

**Affiliations:** 1 Smart Farm Research Center, Korea Institute of Science and Technology (KIST), Gangneung 25451, Republic of Korea; nur.kabadayi@std.yildiz.edu.tr (S.N.K.); noourddin@kist.re.kr (N.B.S.); 2 Department of Plant Science, Gangneung-Wonju National University, Gangneung 25457, Republic of Korea; nipark@gwnu.ac.kr; 3 Department of Botany, Abdul Wali Khan University Mardan, Mardan 23200, Pakistan; hamayun@awkum.edu.pk; 4 Division of Bio-Medical Science and Technology, KIST School, University of Science and Technology (UST), Daejeon 34113, Republic of Korea

**Keywords:** anti-tyrosinase, antioxidant, response surface methodology, *Panax ginseng*, hydroponic, sodium silicate, infrared light

## Abstract

Ginseng has long been widely used for its therapeutic potential. In our current study, we investigated the impact of abiotic stress induced by infrared (IR) radiations and sodium silicate on the upregulation of antioxidant and anti-tyrosinase levels, as well as the total phenolic and total flavonoid contents of the Korean ginseng (*Panax ginseng* C.A. Meyer) variety Yeonpoong. The RSM-based design was used to optimize ultrasonic-assisted extraction time (1–3 h) and temperature (40–60 °C) for better anti-tyrosinase activity and improved antioxidant potential. The optimal extraction results were obtained with a one-hour extraction time, at a temperature of 40 °C, and with a 1.0 mM sodium silicate treatment. We recorded maximum anti-tyrosinase (53.69%) and antioxidant (40.39%) activities when RSM conditions were kept at 875.2 mg GAE/100 g TPC, and 3219.58 mg catechin/100 g. When 1.0 mM sodium silicate was added to the media and extracted at 40 °C for 1 h, the highest total ginsenoside content (368.09 mg/g) was recorded, with variations in individual ginsenosides. Ginsenosides Rb1, Rd, and F2 were significantly affected by extraction temperature, while Rb2 and Rc were influenced by the sodium silicate concentration. Moreover, ginsenoside F2 increased with the sodium silicate treatment, while the Rg3-S content decreased. Interestingly, higher temperatures favored greater ginsenoside diversity while sodium silicate impacted PPD-type ginsenosides. It was observed that the actual experimental values closely matched the predicted values, and this agreement was statistically significant at a 95% confidence level. Our findings suggest that the application of IR irradiation in hydroponic systems can help to improve the quality of ginseng sprouts when supplemented with sodium silicate in hydroponic media. Optimized extraction conditions using ultrasonication can be helpful in improving antioxidant and anti-tyrosinase activity.

## 1. Introduction

*Panax ginseng,* commonly known as Asian ginseng, is a widely recognized traditional medicinal herb that has been used for its remarkable pharmacological properties. Ginseng is renowned for its anti-inflammatory, immunomodulatory, anti-cancer, and ROS-scavenging activities, which are attributed to the presence of its bioactive compounds, such as ginsenosides, polysaccharides, and phenolic compounds. Ginsenosides, the major bioactive compound present in *Panax ginseng*, possess antioxidant and anti-tyrosinase properties, which make them highly valuable in the pharmaceutical and cosmetic industries. However, the content and bioactivity of ginsenosides can vary significantly depending on various factors, such as processing techniques and supplement additives.

Hyperpigmentation is a very common skin condition, caused by the overproduction or overaccumulation of melanin [1]. Tyrosinase is the primary enzyme that plays a crucial role in the synthesis of melanin in mammals by converting L-tyrosine to L-DOPA and forming o-dopaquinone [2]. Overactivity of tyrosinase has been linked to excessive production of melanin pigments, which results in the development of different dermatological disorders such as wrinkles, lentigo, skin aging spots, melasma, nevus, freckles, and melanoma [3]. Although melanin synthesis is a complex process involving multiple steps, it can be controlled by using tyrosinase inhibitors [4]. Tyrosinase inhibitors are substances that can reduce or inhibit the activity of tyrosinase enzymes. Natural inhibitors include hydroquinone, azelaic acid, arbutin, and kojic acid [5]. Plant extracts are a good source of natural anti-tyrosinase agents due to their biochemical structure and serve as a green alternative to synthetic inhibitors [6].

Due to its various advantages, the great demand of the growing world population and increasing environmental challenges, indoor farming systems are becoming more popular in the horticulture industry, and hydroponic farming is one of the promising approaches for reducing the pressure on agricultural land [7,8]. Hydroponics is a modern farming method through which crops are cultivated in nutrient media instead of soil. providing numerous opportunities since it has the ability to better control plants [9]. Water-based nutrient media contain macro- and micronutrient elements with different concentrations, depending on the specific needs of the plant [10]. The quality of nutrient media can be improved by adding different essential elements in media based on re-equipment and uptake of nutrients. Supplementation of the nutrients can be achieved through media modification or by foliar application [11]. Adding fertilizers in media nutrient solution helps to improve the uptake of nutrients by activating the Na/K channel through hairy roots [12]. On the other hand, foliar application allows for the rapid adsorption of essential mineral elements [13]. Fertilizers such as sodium silicate help to improve stomatal conductance and rate of photosynthesis. Recently published studies have shown that foliar application improved overall leaf quality, chlorophyll content, and strength [14].

Artificial lights are one of the most crucial environmental conditions influencing plant development and morphology in indoor farming [15]. Manipulating the quality and quantity of crops to align with retailer and market demands can be achieved through the passive adjustment of factors such as light intensity, photoperiod, and spectrum [16,17]. In many northern greenhouses, supplemental lighting is required from fall through spring to ensure plant growth and development and to produce high-quality plants year-round [18]. The beneficial effects of additional lights, such as different concentrations of blue light supplementation, have been demonstrated in numerous studies showing an increase in total biomass and number of fruits [19]. A study with blue and green light on *Camellia sinensis* reported that light supplementation induced the accumulation of anthocyanins and catechins in the tea plant and can be a good source for industry to enhance the tea quality and taste [20]. According to studies, ginseng quality can be greatly increased in hydroponic systems by using LED lighting [21].

Response surface methodology (RSM) is a multivariate statistical technique that offers a structured and efficient approach to optimization with a relatively small number of experiments [22]. RSM goes beyond the limitations of conventional optimization techniques by considering complex and nonlinear interactions between variables and, due to its flexibility, is a powerful tool for researchers.

In the context of ginseng and its bioactive compounds, the aim of this study is to explore the impact of sodium silicate applied through foliar spray and growth media, coupled with infrared (IR) radiation, on anti-tyrosinase and antioxidant activity. The investigation incorporates response surface methodology (RSM), a powerful multivariate statistical technique, to optimize experimental conditions efficiently.

The novelty of the current study lies in its comprehensive approach to enhancing the bioactivity of ginseng through a synergistic combination of sodium silicate application, IR radiation, and RSM optimization. While previous studies have explored the influence of various factors on ginseng quality, few have investigated the simultaneous effects of sodium silicate, IR radiation, and RSM on anti-tyrosinase and antioxidant activity. This research aims to fill this gap by providing valuable insights into novel strategies for maximizing the therapeutic potential of ginseng for skincare and pharmaceutical applications.

Notable studies in this field include those examining the impact of light conditions on plant development [21,23], the use of hydroponic systems for enhancing ginseng quality with LED lighting [24], and the broader application of RSM in optimizing experimental conditions [25]. However, the integration of these elements in a single study, particularly with a focus on ginseng and its bioactive compounds, represents a distinctive contribution that sets the current work apart from existing research.

The current study aimed to investigate the impact of sodium silicate applied through foliar spray and growth media along with IR radiation on anti-tyrosinase and antioxidant activity. The effectiveness of RSM was sought to be analyzed by manipulating the RSM conditions to achieve optimal results.

## 2. Materials and Methods

### 2.1. Plant Material and Growth Conditions

The Yeonpoong cultivar of Korean ginseng (*Panax ginseng* C. A. Meyer) was acquired from the Korean Ginseng Company, Daejeon, Rp of Korea. Root seedings were incubated at high moisture content for 48 h to provide suitable conditions for the activation of buds. A deep-water culture-based hydroponic setup was designed for cultivation in a vertical farming facility. Environmental conditions were kept constant with a photoperiod of 16 h/8 h (day/night) and a temperature of 24 °C in the day and 18 °C at night. Light intensity was maintained around 65–70 µmol/m^2^/s. RDA (Rural Development Administration)-approved hydroponic nutrient solution was used with little modification in macro- and micronutrients with NO_3_-N 14.5, NH_4_-N 1.5, K 8.5, Ca 4.5, Mg 2.3, PO_4_-P 3.5, SO_4_-S 2.0, Fe-EDTA 0.8, Mn 0.6, B 0.6, Cu 0.02, Mo 0.06, and Zn 0.06 mg/L. The roots of ginseng seedlings were supported with a polyurethane sponge to provide better stability and to float in media. The electrical conductivity of the media was maintained at 2.5 mS/cm with pH 6.8. At the three-prong-leaf stage, seedlings were transferred to the LED chamber for infrared LED exposure treatment [26]. 

### 2.2. Infrared-LED, Sodium Silicate Treatment and Ginsenoside Extraction

For IR treatment, special LED chambers were designed to expose plants for short-term continuous exposure to IR light (760–780 nm). Sodium silicate (Na_2_SiO_3_.9H_2_O) (Pro-TeKt; Dyna-Gro Nutrition Solutions, Richmond, CA, USA) was used as a silicon source that was applied to both aerial parts and supplemented in media of hydroponically grown 2-year-old ginseng seedlings. A complete randomized design was used for this experiment, considering 12 roots per replicate with 5 replicates in total per treatment, including control. A silicate treatment solution was sprayed using hand-spraying bottles, three different concentrations of solution were prepared, i.e., 50, 100 and 200 mM for aerial spraying and for media supplementation. The pH of silicate solution was adjusted to 5.5 ± 0.3 using HCl or NaOH 1 M solution. Treatment lasted for 10 days with a sampling of the whole ginseng plant at the end followed by storage at −80 °C, followed by freeze-drying for 48 h in a freeze dryer (FDCF-12006 Operon Co., Ltd., Gimpo, Republic of Korea). Freeze-dried plant powder was extracted twice with 20 mL of extraction solvent (70% Ethanol) by sonication (UCP02, Jeiotech, Daejeon, Republic of Korea) at three different time and temperature points, i.e., 40, 50, and 60 °C and time ranges from 40, 50, and 60 min. After filtration using filter paper (0.2 μm, ADVANTEC, Dublin, CA, USA), an evaporation method was used to remove the solvent, and the dried residue was dissolved in 100% dimethyl sulfoxide (DMSO) at 40 mg/mL concentration. Each treatment had three biological replicates [27].

### 2.3. Ginsenoside Analysis by HPLC—Evaporative Light Scattering Detection (ELSD)

To determine the ginsenoside content, ginseng extracts were diluted to 20 mg/mL using 100% DMSO. Using a SIL-9A autoinjector, a volume of 10 µL was injected into the HPLC system (Hitachi L-6200 pump, Tokyo, Japan) connected to a Sedex 75 ELSD (Sedere, Vitry-sur-Seine, France) (Shimadzu, Japan). All separations were performed using Agilent Technologies (Palo Alto, CA, USA) Zorbax SB-Aq C18 column (4.6 mm 150 mm, 5 m particle size). The high-pressure liquid chromatographic (HPLC) conditions were as follows: solvent A, water; and solvent B, acetonitrile, 20–22% (0–5 min); 22–25% (5–7 min); 26–30% (27–30 min); 30–35% (30–40 min); 50–70% (45–60 min); 70–85% (60–61 min); and 85–100% (61–90 min). The nebulizer for nitrogen gas was adjusted to 2.5 bar, and ELSD was set to a probe temperature of 75 °C. A total of 5 μg of each ginsenoside standard was injected for HPLC analysis [28]. 

### 2.4. Determination of DPPH Radical Scavenging Activity

Colorimetric analysis based on free radical scavenging activity was performed with the previously reported DPPH decolorization method with few modifications as per experimental requirements [29]. A total of 0.1 mM stock solution of 2,2-diphenyl-1-picryl-hydrazyl (DPPH) was prepared in absolute ethanol. The experiment was performed in a 96-microwell plate setup. Briefly, 190 μL of DPPH was mixed with 10 μL of extracted samples at a starting concentration of 20 mg/mL to 2.5 mg/mL. The reaction mixture was allowed to incubate at room temperature in the absence of light for 30 min, and a multidetection microplate reader (Synergy HT; BioTek Instruments, Winooski, VT, USA) was used to measure absorbance at 517 nm. The percentage of inhibition (%) of DPPH reagent was calculated using the following Equation (1).

(1) Inhibition(DPPH) %=(1−(GE1/BA1))×100

(BA1 is the absorbance of a blank sample (ethanol and DPPH reagent) and GE1 is the absorbance of the tested ginseng extract) [30]. 

### 2.5. Determination of ABTS Radical Scavenging Activity

The antioxidant reagent was freshly prepared before performing the reaction by reacting an equal amount of 7 mM ABTS stock solution with 2.45 mM potassium phosphate solution, and the reaction mixture was incubated at 4 °C in the absence of light to generate ROS. The assay was performed as described previously with little modifications [31]. Ten mg/mL ginseng extract was prepared in DMSO followed by serial dilution, and a total of 190 μL of the ABTS+ reagent was added with 10 μL of GE. The incubation period of 5 min was followed by measurement of absorbance at 734 nm using a microplate reader (Synergy HT; BioTek Instruments, Winooski, VT, USA). The percentage of inhibition (%) of ABTS+ reagent was calculated using the following Equation (2).

(2) Inhibition(ABTS) %=(1−(GE2/BA2))×100

(BA2 is the absorbance of a blank sample (ethanol and DPPH reagent) and GE2 is the absorbance of the tested ginseng extract).

### 2.6. Determination of Total Flavonoid Content (TFC)

Total flavonoids were estimated using the method described by [32]. Briefly, 100 μL of distilled water was mixed with 10 μL of NaNO_2_ solution (5%) and an aliquot (25 µL) of ethanolic extract (10 mg/mL) in triplicates in 96 microwell plates. After 5 min, 15 μL of 10% AlCl_3_ was added to the mixture, which was allowed to stand at room temperature for 6 min, followed by the addition of 50 μL of NaOH solution (1 M) and 50 μL of distilled water. The absorbance of the plate was measured at 510 nm. The total flavonoid content was determined based on the calibration curve of Quercetin (Y = 0.3222x − 0.0021, R_2_ = 0.9979) and expressed in mg of Quercetin equivalent (QE) per 100 g of dry weight of sample.

### 2.7. Determination of Total Phenolic Content (TPC)

The total phenolic content (TPC) in the samples was determined using the Folin–Ciocalten (F-C) method as described by [33] with some modifications. A volume (10 µL) of the ethanolic extract (10 mg/mL) was mixed with 100 µL of sodium carbonate (Na_2_CO_3_, 2%) in a 96 microwell plate. The mixture was incubated in the dark at room temperature for 3 min. This was followed by the addition of 10 µL of 1 N F-C reagent, and the plate was incubated at room temperature under dark for 27 min for color development. The absorbance was measured at 750 nm using a microplate reader (Synergy HT; BioTek Instruments Inc., Winooski, VT, USA). The total phenolic content was determined based on the calibration curve of Gallic Acid (Y = 1.0235x − 0.0002, R_2_ = 0.9968) and expressed in mg of Gallic acid equivalent per 100 g of the dry weight of the sample. 

### 2.8. Tyrosinase Inhibition Activity (Monophenolase)

The monophenolase activity was determined by measuring the hydroxylation of monophenol (L-tyrosine) to dopachrome (a precursor of melanin) [33]. Briefly, 80 μL of 100 mM potassium phosphate buffer (pH 6.8) was prepared in a 96-well plate. Next, 20 μL of test samples in the samples well and solvent in the blank well, and 20 μL of mushroom tyrosinase (1000 units/mL in buffer) were mixed, and PB was used in the blank. Incubate mixture at 37 °C in the dark for 10 min. Eighty μL of 2 mM L-tyrosine solution was added to each well as a substrate, followed by an incubation period of 20 min at 37 °C. The formation of dopachrome was measured by evaluating absorbance at 475 nm using a microplate reader (Synergy HT; BioTek Instruments Inc., Winooski, VT, USA). The relative tyrosinase activity was expressed as % of inhibition using the following Equation (3).

(3) Inhibition(Monophenolase) %=[(D1−E1 /D1]×100

where D1 represents the absorbance in the blank without the inhibitor group and E1 represents the absorbance in the test sample with the inhibitor included.

### 2.9. Tyrosinase Inhibition Activity (Diphenolase)

Oxidation of L-DOPA (o-diphenol) was used to measure the tyrosinase inhibition activity of ginseng extract. Following the protocol mentioned [34], with few modifications, 80 μL of 100 mM potassium phosphate buffer (pH 6.8), 20 μL of extract solvent, and 20 μL of mushroom tyrosinase (1000 units/mL in buffer) were incubated for 10 min at 37 °C. After adding 80 μL of 4 mM L-DOPA solution to each well as a substrate, the reaction was performed at 37 C in the dark. The relative tyrosinase activity was expressed as a percentage of inhibition using the following Equation (4).

(4) Inhibition(Diphenolase) %=[(D2−E2 /D2]×100

where D2 represents the absorbance in the blank without inhibitor group and E2 represents the absorbance in the test sample with the inhibitor included.

### 2.10. Experimental Design (RSM Model)

Extraction optimization for higher total ginsenoside content, anti-tyrosinase potential, and antioxidants from IR-treated, sodium silicate-supplemented hydroponic ginseng was carried out using response surface methodology (RSM). A five-level, two-variable central composite design (CCD) was employed to investigate the best combination of duration and temperature of the extraction process among all three sodium silicate concentration treatments. Extraction duration (Hours, X1) and extraction temperature (C, X2) were used as independent variables. The range and central point were decided based on a pre-experiment investigation conducted before performing the main experiment. Twenty-seven factorial points, including five central points, were used in the CCD model. All experiments were performed in triplicate order and average values were considered. The best condition for response was selected as the response of a combination of independent variables. Response surface regression was used to fit the second-order polynomial model. Equation (5).

(5) Y=β0+ΣβiXi+ΣβiiX2i+ΣβijXiXj

where Y is the predicted response, β_0_ is an intercept, β_i_, β_ii_, and β_ij_ are the coefficients of the linear, quadratic, and interaction terms, respectively. X_i_ and X_j_ are coded independent variables. Design Expert version 12.0.3 software (Stat-Ease Inc., Minneapolis, MN 55413, USA) was used for the RSM model investigation. The Fisher test value (*F*-value) from the ANOVA test, coefficient of determination (R^2^), and lack of fit were used to check acceptance of the model. The difference was considered significant at *p* < 0.05.

### 2.11. Statistical Analysis

All data are expressed as the mean ± standard deviation (SD) for three independent replicates used in the experiments. The statistical data were analyzed using the GraphPad Prism 8 software package (GRAPH PAD software Inc., La Jolla, CA, USA). For comparison studies, one-way ANOVA with post hoc Tukey’s test was performed. The statistical significance was set at *p* < 0.05.

## 3. Results and Discussion

### 3.1. Impact of IR Treatment on Ginsenosides Content and Extraction Yield

**Figure 1:**
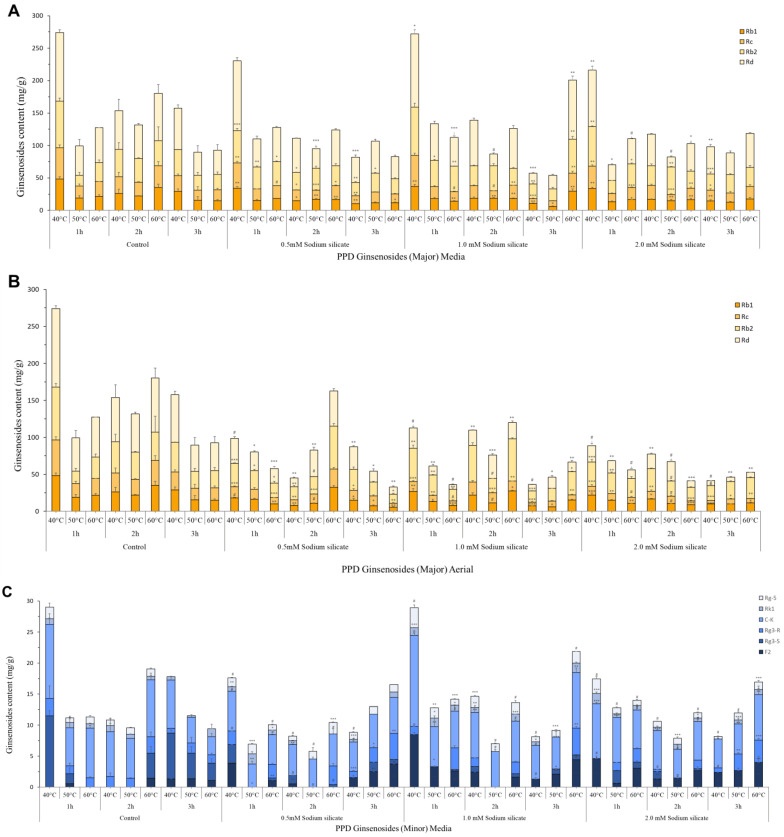

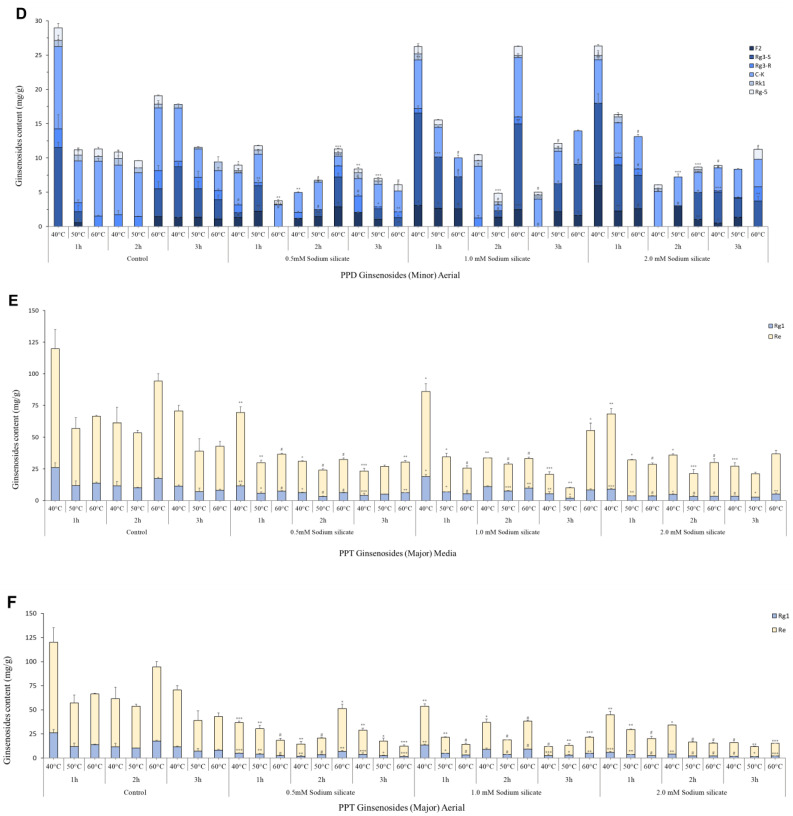
Effect of different concentrations of sodium silicate under IR treatment on extraction of ginsenosides at different time and temperature. (**A**,**D**) PPD-type major ginsenosides in media and aerial treatment. (**B**,**E**) PPD type minor ginsenosides in media and aerial treatment. (**C**,**F**) PPT-type major ginsenosides in media and aerial treatment. Asterisks (*) indicate significant differences between control and different elicitor treatments (*p* < 0.05, Student’s *t*-test). * *p* < 0.05, ** *p* < 0.01, *** *p* < 0.001, # *p* < 0.0001, ns stands for not significant compared to the control group.

**Figure 2:**
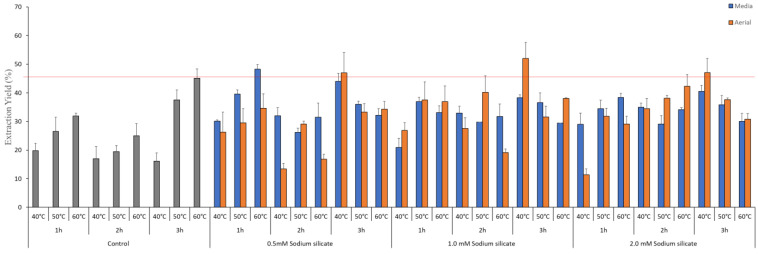
Extraction yield (%) of media and aerial treatment of freeze-dried ginseng with different sodium silicate concentrations, extraction time and temperature.

HPLC system connected to ELSD was used to identify and quantify various ginsenosides by comparing the retention times of the detected with those of the pure compounds. Twelve ginsenosides were detected and quantified using standards i.e., Rb1, Rc, Rb2, Rd, F2, Rg3-S, Rg3-R, C-K, Rk1, Rg1, Re and Rg5. The calibration curve of standards used is elaborated in Figure S1, and the TIC of control, aerial, and media treatment is given in Figure S2. The results of ginsenoside content in IR-treated silicate-supplemented hydroponic ginseng are shown in Figure 1. Elicitation of hydroponic media with IR light and sodium silicate both in media and spraying on the aerial part of the plant affects not only total ginsenoside content in all extraction conditions but also minor ginsenoside content were also significantly improved. Sodium silicate is known to enhance secondary metabolite production in plants through various mechanisms, including improved nutrient uptake and stress response [35]. Sodium silicate had a dramatic effect when added to hydroponic media alongside IR exposure. IR appears to function as an elicitor, prompting ginseng plants to enhance ginsenoside production. This response can be interpreted as a defense mechanism, as plants often increase the synthesis of secondary metabolites in response to environmental stressors [36]. Sodium silicate effectively improved ginsenoside content when elicitation was near the root zone [37]. In media treatment, 1.0 mM treatment of sodium silicate extracted for 1 h at 40 °C showed maximum overall total ginsenoside content with a total ginsenoside content of 368.09 ± 13.52 mg/g followed by 0.5 mM and 2.0 mM sodium silicate treatments with ginsenoside content being 308.75 ± 22.4 and 289.03 ± 21.45 mg/g. The impact of IR treatment and application of sodium silicate as a supplement both in hydroponic media and aerial spraying on leaves shows a different concentration of protopanaxadiol (PPD) and protopanaxatriol (PPT) type ginsenosides, as shown in Figure 1. A total of 12 ginsenosides, including 10 PPD (Rb1, Rc, Rb2, Rd, F2, Rg3-S, Rg3-R, C-K, Rk1, and Rg5) type and 2 PPT (Rg1, Re) type ginsenosides in extract from treated plants, were quantified by comparison of retention time using standards. PPD ginsenosides include four major ginsenosides and six minor ginsenosides based on quantity. Ginsenoside Rb1 showed sensitivity against the extraction temperature in both aerial and media treatment. The lower extraction temperature showed a higher content of Rb1, and at 1 h 40 °C extraction point a higher Rb1 content was observed in the control treatment (48.403 ± 3.53 mg/g) followed by a 1.0 mM sodium silicate treatment extracted (36.76 ± 2.41 mg/g). These findings align with previous studies on ginsenoside biosynthesis, which show that the regulation of specific ginsenoside types can be influenced by external factors [38]. Rd ginsenoside was found to be the most abundant in media supplementation treatment of sodium silicate, whereas ginsenoside Rb2 was overall higher in aerial spray treatment. Ginsenoside Rc contents were getting significantly higher with an increase in concentration of sodium silicate treatment, with a 1.0 mM sodium silicate treatment being the most effective treatment. Higher extraction time and temperature have a negative impact on Rc content. Figure 1B,E represents the content of individual minor ginsenosides in both aerial and media treatments. The content of ginsenoside F2 was reported to be positively correlated to the sodium silicate treatment followed by the extraction condition. The most effective was a 1.0 mM sodium silicate treatment in terms of ginsenoside F2 content. The content of ginsenoside Rg3-S was inversely proportional to sodium silicate treatment, whereas Rg3-R showed an opposite trend in media-supplemented treatment. C-K was the most abundant minor ginsenoside in media treatment. This is consistent with research that indicates ginsenoside transformation and bioconversion can be temperature-dependent processes [39]. Aerial spraying treatment of sodium silicate expressed the same response of ginsenoside F2 as in media treatment, suggesting that the sodium silicate treatment helps to upregulate factors that are responsible for the conversion of ginsenoside Rd to F2. This is supported by the observed increase in ginsenoside F2 content with sodium silicate treatment, as F2 is a product of the bioconversion of Rd [40]. In aerial treatment, ginsenoside Rg3-S was the most abundant ginsenoside, followed by C-K. Of the two isomers, i.e., Rg3-S and Rg3-R, one was predominant in media treatment i.e., ginsenoside Rg3-R, whereas Rg3-S was significantly higher in aerial treatment. Ginsenosides Rg5 and Rk1 were higher in media treatment but not significantly different than content in aerial treatment. When it comes to the overall composition of ginsenosides, maximum diversity was observed at a 40 °C extraction temperature, and higher contents were detected when extracted for higher temperature conditions. This highlights the influence of cultivation methods on ginsenoside composition, especially for elicitation [41]. Among PPT-type ginsenoside (Figure 1C,F), both aerial and media supplementation treatment showed higher content of ginsenoside Re in the control treatment, in which there was no significant impact of the sodium silicate treatment on PPT-type ginsenosides, suggesting that sodium silicate affects mostly pathways linked to PPD type ginsenoside bioconversion. PPT-type ginsenosides are sensitive to extraction temperature conditions as there was a significant drop in content when extraction, the temperature and duration were increased. The extraction yield was positively correlated to extraction duration and time. This emphasizes the need to carefully control extraction conditions to preserve PPT-type ginsenosides [42]. There was no significant difference in the extraction yield of the sodium silicate treatment when compared to the control (Figure 2).

### 3.2. Effect of Sodium Silicate Treatment under IR LED on Antioxidant Potential

The effect of different extraction time points on the scavenging potential of IR-treated hydroponic ginseng supplemented with sodium silicate was examined against stable free radicals such as DPPH and ABTS. The various concentrations of extracts optimized for scavenging DPPH free radicals were 125, 250, 500, and 1000 µg/mL, and 62.5, 125, 250, and 500 µg/mL for ABTS. For DPPH radical scavenging assay, 1.0 mM sodium silicate-treated plant in media supplementation extracted at 50 °C for 1 h exhibited the highest percentage of scavenging potential with 800.96 ± 6.69 μg/mL RC_50_ when compared to control and aerial treatment. Previous studies have also reported the impact of extraction conditions on the antioxidant activity of plant extracts. For instance, a study by Choi et al. (2022) [43] demonstrated that the temperature and time of extraction can influence the antioxidant capacity of ginseng extracts. According to the RC_50_ (μg/mL) findings presented in Table 1, the DPPH free radical scavenging assay demonstrated the ability of the sodium silicate treatment under IR irradiation to effectively scavenge free radicals. At a concentration of 1000 μg/mL IR-treated hydroponic ginseng supplemented with 1.0 mM sodium silicate, extracted at 50 °C, 1 h showed the lowest inhibition percentage of 94.65 ± 1.02% in aerial spray treatment followed by a 2.0 mM concentration extracted at 50 °C for 1 h with an inhibition percentage value of 90.19 ± 1.58%. Maximum inhibition percentage for DPPH scavenging activity in media-supplemented treatment was observed in 1.0 mM concentration extracted at 1 h 40 °C, 57.68 ± 4.27%. The results from Table 2, indicate that the sodium silicate treatment, coupled with infrared (IR) irradiation, exhibited notable efficacy in scavenging free radicals, as demonstrated by the RC50 (μg/mL) values in the ABTS free radical scavenging assay.

### 3.3. Impact on Total Phenolic and Flavonoid Content

**Figure 3:**
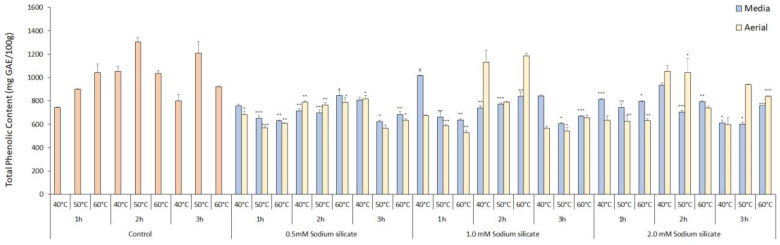
Total phenolic content of IR-supplemented sodium silicate-treated *Panax ginseng* at different extraction time points and temperatures in mg GAE/100 g. Data are presented as means ± SD from three replicates; * *p* < 0.05, ** *p* < 0.01, *** *p* < 0.001, # *p* < 0.0001 compared to the control group.

**Figure 4:**
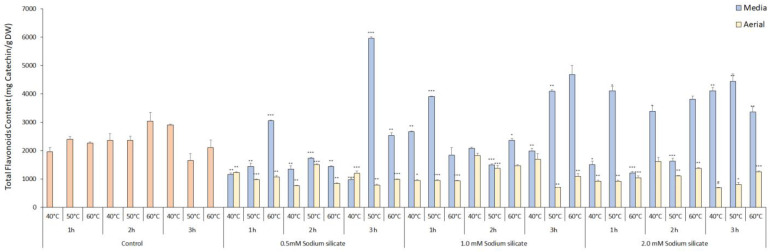
Total flavonoid content of IR-supplemented sodium silicate-treated *Panax ginseng* at different extraction time points and temperatures in mg catechin/DW. Data are presented as means ± SD from three replicates; * *p* < 0.05, ** *p* < 0.01, *** *p* < 0.001, # *p* < 0.0001 compared to the control group.

The TPC levels in both the media- and the foliage-sprayed treatments showed a negative correlation with the application of sodium silicate, compared to the control (Figure 3). The presence of silicate either by uptake via stomata or by hair roots in the media system has a negative impact on the biosynthesis of phenolic compounds, which may be attributed to higher osmotic potential generating a slight salinity condition resulting in a decrease in total phenolic content [44]. The control treatment exhibited the highest amount of phenolics at 2 h and 50 °C, measuring 1304.02 ± 36.84 mg GAE/100 g, followed by the control treatment at 3 h and 50 °C, which measured 1208.90 ± 100.35 mg GAE/100 g. The duration of extraction and temperature had a significantly positive effect on the phenolics content, with the optimal conditions being 2 h for extraction time and 50 °C for temperature in control. The total phenol content (TPC) yield demonstrated an initial increase with rising extraction temperature and prolonged extraction time. However, this positive trend was followed by a subsequent decrease in TPC yield. This phenomenon can be attributed to the breakdown of the plant cell wall, facilitated by water’s role as a swelling agent, leading to enhanced extraction of phenolic compounds. The solvent ethanol aids in dissolving the polyphenols by disrupting the solid–solvent contact area, further enhancing the extraction process [45]. Ultrasonication has shown similar results when investigated against conventional extraction methods in food processing [46]. There were slight variations in content between the aerial and media application of sodium silicate within the treatments. Sodium silicate 1.0 mM, 2 h 60 °C in aerial showed the best results, whereas sodium silicate 1.0 mM 1 h 40 °C was the best for media treatment in sodium silicate-treated extracts with the content of 1183.52 ± 23.02 and 1017.42 ± 4.605 mg GAE/100 g, respectively. It was previously reported that the sodium silicate treatment enhances the total phenolic content when treated with Fe-EDTA and sodium [47,48]. Overall, phenolic content remained stable across different temperatures and time points. On the other hand, the total flavonoid content increased proportionally with extraction time in the media treatment, with the highest content observed in the sodium silicate 0.5 mM, 3 h, and 50 °C treatment, measuring 5966.27 ± 43.89 mg catechin/g DW (Figure 4). In aerial spraying, the control treatment with 2 h and 60 °C extraction condition showed the best result for total flavonoid content with 3045.39 ± 307.24 mg catechin/g DW. Total flavonoid contents tend to increase with an increase in time for extraction due to a decrease in the viscidity of solvent with an increase in temperature, resulting in the release of bioactive compounds from plant cells. Increasing the temperature higher than the optimum extraction point specific for each plant material results in a decrease in total flavonoid contents [49]. 

### 3.4. Tyrosinase Inhibition Potential 

Melanin is a natural pigment found in various living organisms and is responsible for determining the color of our skin, hair, and eyes. Its production involves several enzymatic and nonenzymatic steps leading to different types of melanin [50]. The process starts with the conversion of the amino acid tyrosine to L-DOPA by the enzyme tyrosinase. Subsequent enzymatic oxidations convert L-DOPA into L-dopaquinone, which then undergoes polymerization, joining together to form larger molecules of melanin. In cosmetic products for skin whitening, the inhibition of tyrosinase activity is crucial [51]. By reducing tyrosinase’s action, the formation of L-DOPA and L-dopaquinone, and subsequently, melanin polymerization is slowed down [52]. As a result, the overall production of melanin is reduced, leading to a lighter and more even skin tone and addressing hyperpigmentation issues effectively.

#### 3.4.1. Monophenolase Inhibition 

**Figure 5:**
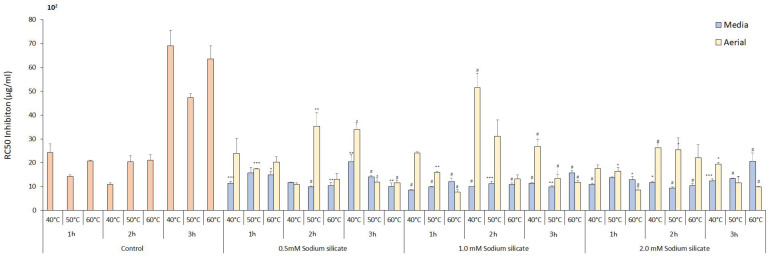
RC_50_ inhibition (µg/mL) of monophenolase activity of ginseng. Data are presented as means ± SD from three replicates; * *p* < 0.05, ** *p* < 0.01, *** *p* < 0.001, # *p* < 0.0001 compared to the control group.

Monophenolase activity is responsible for the oxidation of monophenols to o-diphenols. This activity is critical for melanin synthesis, the pigment responsible for skin, hair, and eye color. Inhibition of monophenolase activity can have therapeutic potential in conditions characterized by excessive melanin production, such as hyperpigmentation disorders (e.g., melasma) and certain types of freckles. By inhibiting this activity, it is possible to reduce melanin production and subsequently lighten skin tone [53]. The influence of sodium silicate supplementation with IR treatment on the anti-tyrosinase potential of *Panax ginseng* was investigated using 2 mM L-Tyrosine as the substrate. The results demonstrated a dose-dependent inhibition of melanogenesis, as depicted in Figure 5. Particularly, the treatment with 1.0 mM sodium silicate led to the rapid conversion of mono-phenols to o-diphenolase after 1 h of extraction time at 40 °C in the media. The effectiveness of sodium silicate can be explained by the fact that tyrosinase needs to be converted to oxy form after passing a lag time phase, during which the met form of tyrosinase only converts o-diphenols into o-quinones [54]. Moreover, when the extraction process involved sodium silicate at a concentration of 1.0 mM and was conducted for 1 h at 60 °C, it exhibited the highest level of inhibition in the samples subjected to aerial treatment. The trend in media supplementation was not significantly different among the different concentrations of the sodium silicate treatment at various extraction time points. Meanwhile, in the control treatment, increasing extraction duration inversely influences the inhibition potential both in media and aerial treatments. The decrease in inhibition potential of control samples can be attributed to a reduction in flavonoid contents with an increase in extraction duration, which has been previously reported [55]. In aerial treatment, a higher extraction temperature was more effective against the inhibition of monophenolase enzyme when compared to other extraction temperatures and controls. Aerial treatment tends to have a higher content of ginsenoside Re and minor ginsenoside Rg3-S at higher extraction temperature points which are previously reported to have higher anti-tyrosinase potential [56,57]. Sodium silicate treatment improved monophenolase activity compared to control 8.6-fold in media, whereas it was an 8.89-fold improvement in aerial treatment. Overall, control treatment showed significantly lower inhibition activity compared to the sodium silicate treatment and the sodium silicate 1.0 mM concentration performed best among the other concentrations.

#### 3.4.2. Diphenolase Inhibition 

**Figure 6:**
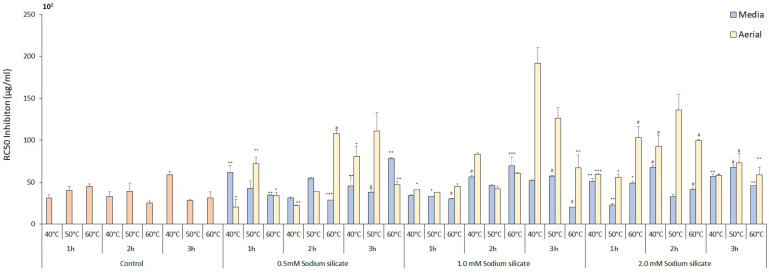
RC_50_ inhibition (µg/mL) of diphenolase activity of ginseng. Data are presented as means ± SD from three replicates; * *p* < 0.05, ** *p* < 0.01, *** *p* < 0.001, # *p* < 0.0001 compared to the control group.

In this study, the inhibitory effect of sodium silicate on the diphenolase activity of mushroom tyrosinase for the oxidation of L-DOPA was investigated (Figure 6). Diphenolase inhibitors work by inhibiting the diphenolase activity of tyrosinase. It chelates copper at the active site of the enzyme and competes with the substrate for the active site, thereby inhibiting the enzyme activity [58]. For media treatment, the best inhibition was shown by the sodium silicate treatment of 1.0 mM concentration extracted for 3 h at 60 °C, which was the highest extraction time and temperature points for our experiment, consistent with previous research on enzyme inhibition [59]. However, for aerial treatment, the best treatment was shown at the right opposite extraction conditions with the sodium silicate treatment of 0.5 mM and 1 h 40 °C setup. Our results showed that the sodium silicate treatment improved the inhibition activity 2.90- and 2.88-fold better compared to the control treatment in media and aerial treatment, respectively. The inhibition strength follows the order: 1 h 40 °C aerial 0.5 mM > 2 h 40 °C aerial 0.5 mM 3 h 60 °C media 1.0 mM > 1 h 50 °C media 2.0 mM. In the media treatment, 1 and 2 h extraction time points at 40 °C lower concentrations of sodium silicate were more effective; and at 50 °C, the highest concentration of 2.0 mM was performing better within the treatments. These results are consistent with previous literature on the sensitivity of enzyme inhibition to factors such as temperature and concentration [60]. In control treatment compared to sodium silicate, 2–3 h extraction duration tends to perform better inhibition activity of tyrosinase enzyme. In the case of the aerial treatment of sodium silicate, a 3 h extraction time decreased the inhibitory effect on the diphenolase activity. In 1 and 2 h extracted samples, lower concentrations of sodium silicate worked better than the control treatment. Three h of extraction time gave a better inhibition percentage without the sodium silicate treatment and affected the inhibition potential of the sodium silicate-treated samples. It can be concluded from the results that the inhibition potential of the sodium silicate treatment on the diphenolase activity of the enzyme is sensitive to sodium silicate concentrations, and when supplemented in hydroponic media, it gives good results when compared to aerial spray treatment. Ref. [61] reported that *P. ginseng* leaf-capped gold nanoparticles exhibit good inhibitory potential against the diphenolase enzyme and [62] reported that minor ginsenoside plays an important role in the inhibition of the tyrosinase enzyme-related pathway. 

### 3.5. Extraction Optimization

#### 3.5.1. Analysis and Fitting of Model

The response surface methodology was applied to optimize the extraction of TFC, TPC, antioxidant activity, anti-tyrosinase activity, extraction yield, and total ginsenoside content from hydroponic ginseng. Observed values of all dependent variables (antioxidant, TPC, TFC, anti-tyrosinase, and extraction yield) and the independent variables (ultrasound extraction temperature and duration of extraction) obtained from three different concentrations of sodium silicate, i.e., 0.5, 1.0, and 2.0 mM, central composite design of two factors with their observed responses using RSM are shown in Table 3. A total of 1.0 mM sodium silicate in media supplementation was selected for optimization based on the relatively higher TPC, and TFC content with lower RC_50_ values of antioxidant and higher inhibition for both monophenolase and diphenoloase activity. Whereas 0.5 mM expressed intermedia results among sodium silicate treatments. Similarly, the equations below represent the second-order full polynomial models with regression coefficients used to predict the dependent variable, where X_1_ and X_2_ are independent variables for ultrasound extraction time, and duration of extraction, respectively. The regression coefficients, R^2^, and lack of fit of the obtained second-order polynomial equation for the various responses of sodium silicate-treated hydroponic ginseng are shown in Table 4.

(6) $$\begin{array}{l} Antioxidant=69.3161-2.6664X_{1}-19.3747X_{2}+0.8507X_{1}X_{2}+0.0281{X_{1}}^{2}-\\ 1.7775{X_{2}}^{2}-0.0099{X_{1}}^{2}X_{2}+0.0465X_{1}{X_{2}}^{2} \end{array}$$

(7) $$\begin{array}{l} TPC=111.88-2.1418X_{1}-66.1734X_{2}+1.4644X_{1}X_{2}+0.0079{X_{1}}^{2}+\\ 14.4628{X_{2}}^{2}-0.0014{X_{1}}^{2}X_{2}-0.3084X_{1}{X_{2}}^{2} \end{array}$$

(8) $$\begin{array}{l} TFC=235.08-4.8340X_{1}-84.1827X_{2}+0.8162X_{1}X_{2}+0.0344{X_{1}}^{2}+\\ 11.4843{X_{2}}^{2} \end{array}$$

(9) $$\begin{array}{l} Anti-tyrosinase=41.9814-1.1930X_{1}-21.1115X_{2}+0.6239X_{1}X_{2}+\\ 0.0100{X_{1}}^{2}+2.5223{X_{2}}^{2}-0.0043{X_{1}}^{2}X_{2}+0.0461X_{1}{X_{2}}^{2} \end{array}$$

(10) $$\begin{array}{l} ExtractionYield=-17.35+0.7956X_{1}+12.6866X_{2}-0.4353X_{1}X_{2}-0.0064{X_{1}}^{2}-\\ 0.5010{X_{2}}^{2}+0.0033{X_{1}}^{2}X_{2}-0.0141X_{1}{X_{2}}^{2} \end{array}$$

As per Table 4, the *p* values of the lack of fit indicate that the variables analyzed were sufficient to explain the proposed model. R2 values of antioxidant, TPC, TFC, anti-tyrosinase, and extraction yield were 0.99, 0.95, 0.96, 0.93, 0.89, and 0.91, respectively, the lower values of statistical parameters for the responses, confirming the desirability of the generated model. All five responses exhibited a nonsignificant lack of fit, with values of 7.38, 34.39, 70.13, 1.33, and 0.56, respectively, indicating that the models fit the data well. Thus, the model was appropriate for predicting the best extraction conditions within the tested range.

#### 3.5.2. Effect of Time on Antioxidant, Anti-Tyrosinase, TPC, TFC and Extraction Yield 

**Figure 7:**
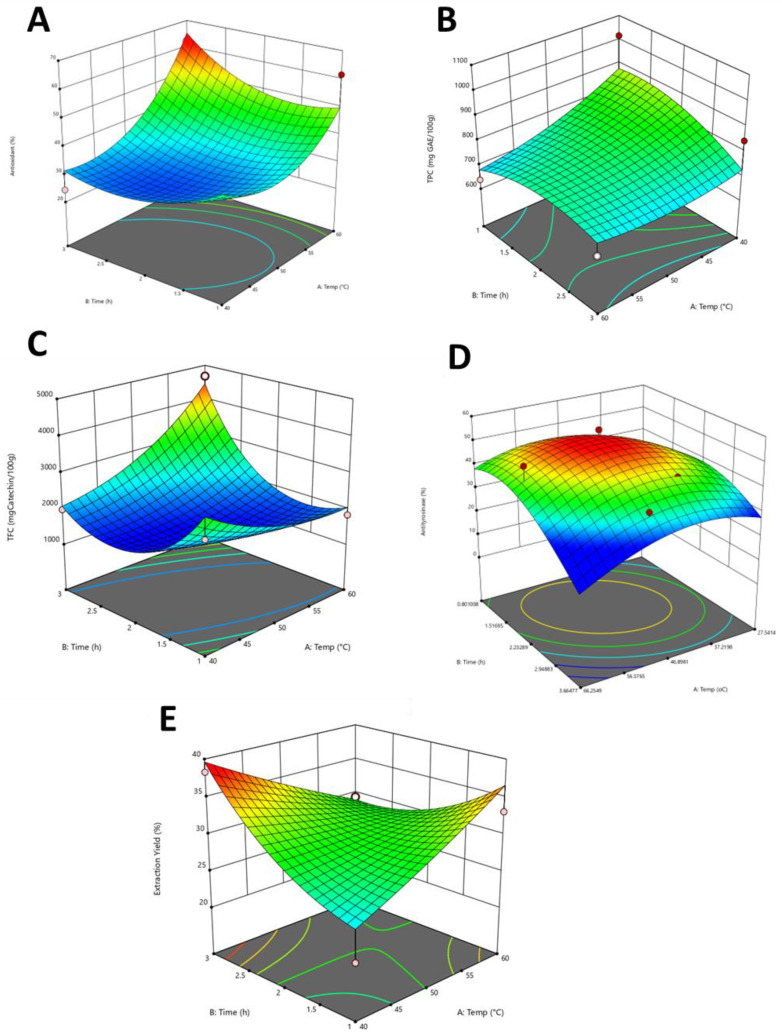
Response surface plots of (**A**) time/temperature on antioxidant potential (quadratic model); (**B**) time/temperature on total phenolic content (TPC quadratic model); (**C**) time/temperature on total flavonoids content (TFC); (**D**) time/temperature on anti-tyrosinase potential; (**E**) time/temperature on extraction yield.

The extraction time of sonication showed a highly significant impact on extraction of TPC, TFC, extraction yield, and on performed biological activities, such as antioxidant and tyrosinase inhibition activity. The observed quadratic positive effect of extraction time and temperature on the antioxidant potential of ginseng extract aligns with the research by Choi et al. (2022) [43]. The time of extraction is expressed in quadratic terms as X_1_ and X_1_^2^ for sodium silicate 0.5, 0.1, and 2.0 mM concentration treatment. The temperature was expressed as X_2_ in linear and X_2_^2^ in quadratic expression. It was reported that the quadratic effect of time (X_1_) and temperature (X_2_) was positive and significant at *p* < 0.01. Figure 7A expresses the impact of extraction time and temperature on the antioxidant potential of ginseng extract supplemented with 1.0 mM sodium silicate, the region of 60 °C and 3 h extraction time would achieve a higher inhibition of antioxidant potential (64.88%), whereas the lowest values obtained were 24.45% at 45.7 °C and 2 h extraction time point. The mean values and ANOVA data are presented in Table 4. Antioxidant activity was positively affected by extraction time and temperature. The three-dimensional RSM plot in Figure 7A represents the interaction between extraction time and temperature. The mean response values of TPC are expressed in Table 3. The highest value of 880.44 mg GAE/100 g was obtained at 1 h 30 min extracted at 40 °C, whereas the value 677.63 mg GAE/100 g was obtained at 1 h and 60 °C. Temperature (X_1_) (*p* < 0.001) and treatment time (X_2_) (*p* < 0.001) exhibited a linear significant negative effect towards extraction of TPC. Similarly, the interaction term X_1_X_2_ (*p* < 0.05) and each quadratic term substantially impacted the TPC yield. Our findings that longer extraction times and lower temperatures are associated with higher TPC values are consistent with the work of Yao et al. (2015) [63]. The three-dimensional surface plots shown in Figure 7B; describe the interaction between temperature (X_1_) and treatment time (X_2_). The highest TFC value of 4446.86 mg catechin/g DW was achieved at a treatment time of 3 h and a 60 °C extraction temperature, whereas the lowest value, 1491.83 mg catechin/g DW, was obtained at 2 h and 44 °C extraction time and temperature, respectively. Total phenolic content shows a linearly positive significance response to extraction temperature (X_1_) and time of extraction (X_2_), which shows an increase in the value of TFC with the increase in extraction duration and temperature. The relationship between extraction time, temperature, and TFC aligns with the study conducted by Malathy et al. (2020) [64], which reported that extended extraction times and higher temperatures positively affected the TFC in ginseng extracts. Our results confirm this trend, with the highest TFC value observed at 60 °C and a 3 h extraction time. The three-dimensional surface plot is represented in Figure 7C. The mean value of the extraction yield is presented in Table 3. The highest value of 38.53% was obtained at a 41.11 °C temperature and 2.97 h time treatment, whereas the lowest extraction yield of 25.01% was obtained at a treatment time of 1 h and an extraction temperature of 40 °C. The complex response of extraction yield to time and temperature is well elaborated by Dzah et al. (2020). Longer extraction times increased yield due to better compound solubilization and excessively high temperatures degrading certain bioactive compounds and reducing overall yield [29]. The ANOVA of the data represents a positive effect of linear terms, i.e., treatment time (X_2_) (*p* < 0.0001) and a negative effect on interaction terms such as temperature. Increasing extraction time has a positive impact on extraction yield, but increasing extraction temperature has a negative impact. 

#### 3.5.3. Validation of Results 

To verify the predictive ability of the model, experimental and predictive responses for 1 h and 40 °C for 1.0 mM sodium silicate in media supplementation were compared. Under the mentioned extraction conditions, predicted and observed values of antioxidant activity were 64.88% and 62.08%, TPC was 880.45 mg GAE/100 g and 1017.42 mg GAE/100 g, TFC was 4446.86 mg catechin/g DW and 4683.43 mg catechin/g DW, tyrosinase inhibition potential was 56.87% and 53.69%, and the extraction yields were 38.53% and 38.3%, respectively. The predicted values have shown little nonsignificant difference when compared to observed values, validating the suitability of the fitted RSM model. 

## 4. Conclusions

The experimental results demonstrated that different treatment conditions significantly affected the ginsenoside content, extraction yield, anti-tyrosinase, and antioxidant composition of the plant extract. These findings could be used for the development of optimized extraction protocols for obtaining plant extracts with enhanced bioactive compounds and antioxidant and anti-tyrosinase activity. Further studies should be conducted to explore the underlying mechanisms and potential applications of these plant extracts in the food, pharmaceutical, and cosmetic industries.

## Figures and Tables

**Table 1:** DPPH assay based on free radicals scavenging potential expressed as RC_50_ values of media- and aerial-treated samples.

		Media Treatment DPPH RC_50_ (µg/mL ± SD)				Aerial Treatment DPPH RC_50_ (µg/mL ± SD)		
Time	Temperature	Control	0.5 mM	1.0 mM	2.0 mM	0.5 mM	1.0 mM	2.0 mM
1 h	40 °C	1679.10 ± 50.04	864.88 ± 1.07 ***	823.53 ± 72.50 ***	893.26 ± 6.94 ***	509.56 ± 0.06 ***	445.00 ± 5.42 ***	482.66 ± 4.56 ***
	50 °C	1538.80 ± 17.16	886.88 ± 3.29 #	800.96 ± 6.69 #	928.17 ± 9.74 #	479.91 ± 7.27 #	462.21 ± 0.89 #	484.59 ± 1.76 #
	60 °C	1359.33 ± 22.90	920.96 ± 2.18 #	907.53 ± 10.21 #	931.20 ± 4.20 #	498.55 ± 2.92 #	1004.11 ± 38.84 #	1017.08 ± 35.80 ***
2 h	40 °C	975.88 ± 54.90	867.60 ± 17.10 *	1138.06 ± 1.94 **	1030.48 ± 1.55 ns	503.74 ± 2.97 #	917.58 ± 5.79 ns	827.45 ± 8.62 **
	50 °C	891.74 ± 16.40	1042.99 ± 5.76 #	1051.75 ± 17.40 #	1106.32 ± 9.56 #	860.60 ± 1.04 ns	894.31 ± 15.34 ns	975.42 ± 44.71 ns
	60 °C	824.25 ± 5.80	1352.92 ± 11.53 ***	933.89 ± 5.92 **	1102.19 ± 4.86 **	896.12 ± 9.58 ns	931.50 ± 12.21 ns	919.11 ± 9.25 #
3 h	40 °C	824.25 ± 5.80	1172.51 ± 51.14 #	1143.95 ± 54.21 #	899.53 ± 12.29 #	857.8 ± 8.65 ***	430.53 ± 31.05 #	1009.29 ± 10.83 #
	50 °C	804.08 ± 2.72	889.10 ± 1.26 ***	861.86 ± 10.21 ***	944.47 ± 18.24 **	911.22 ± 25.60 *	936.31 ± 1.46 **	1037.65 ± 31.50 ns
	60 °C	1005.08 ± 37.18	956.56 ± 3.86 *	950.82 ± 7.16 #	1211.15 ± 5.75 **	984.21 ± 13.77 ***	785.62 ± 15.90 **	1021.07 ± 38.68 ***

Data are presented as means ± SD from three replicates; * *p* < 0.05, ** *p* < 0.01, *** *p* < 0.001, # *p* < 0.0001, ns stands for not significant compared to the control group.

**Table 2:** ABTS assay-based free radicals scavenging potential expressed as RC_50_ values of media- and aerial-treated samples.

		Media Treatment ABTS RC_50_ (µg/mL ± SD)				Aerial Treatment ABTS RC_50_ (µg/mL ± SD)		
Time	Temperature	Control	0.5 mM	1.0 mM	2.0 mM	0.5 mM	1.0 mM	2.0 mM
1 h	40 °C	796.14 ± 73.14	682.18 ± 17.81 *	582.38 ± 9.76 **	625.20 ± 33.08 **	892.02 ± 38.13 *	588.20 ± 24.75 **	752.24 ± 27.32 ns
	50 °C	1141.39 ± 45.71	1054.93 ± 17.18 **	1078.75 ± 39.37 **	1012.84 ± 21.41 ns	846.23 ± 33.00 **	947.82 ± 52.75 **	822.01 ± 29.51 ***
	60 °C	785.94 ± 44.15	1035.91 ± 58.23 *	80.95 ± 5.84 **	941.42 ± 93.80 #	1051.28 ± 8.83 ***	97.80 ± 15.72 #	1611.01 ± 82.97 **
2 h	40 °C	1042.89 ± 61.99	875.03 ± 10.78 ns	904.15 ± 59.03 **	867.10 ± 5.87 *	1084.95 ± 63.31 ns	717.83 ± 67.71 **	912.49 ± 2.90 **
	50 °C	931.09 ± 62.62	1001.03 ± 51.38 ns	1215.46 ± 14.11 ns	2307.35 ± 81.25 ***	1042.32 ± 46.41 ns	1088.98 ± 44.21 *	1101.60 ± 65.82 *
	60 °C	737.83 ± 31.83	1137.37 ± 60.57 #	123.57 ± 20.09 **	1205.47 ± 41.30 ***	821.52 ± 9.71 ns	517.94 ± 12.23 *	1062.44 ± 53.13 *
3 h	40 °C	1879.36 ± 70.01	1048.18 ± 90.28 ***	836.68 ± 39.58 ns	338.10 ± 39.71 **	1354.31 ± 55.41 **	983.55 ± 6.13 ***	1299.70 ± 32.39 **
	50 °C	602.39 ± 6.13	244.79 ± 2.61 ***	429.99 ± 31.64 #	293.32 ± 25.45 #	1035.12 ± 32.22 #	1013.87 ± 6.81 #	912.59 ± 45.89 #
	60 °C	943.84 ± 22.95	180.35 ± 1.65 #	104.69 ± 10.94 #	53.48 ± 4.75 #	962.75 ± 43.58 *	134.07 ± 2.31 #	812.93 ± 67.47 *

Data are presented as means ± SD from three replicates; * *p* < 0.05, ** *p* < 0.01, *** *p* < 0.001, # *p* < 0.0001 ns stands for not significant compared to the control group.

**Table 3:** Central composite design of two factors with their observed responses using RSM.

Run	X1	X2	Antioxidant %			TPC mg GAE/100 g			TFC mg Catechin/100 g			Anti-Tyrosinase %			Yield %		
			0.5 mM	1.0 mM	2.0 mM	0.5 mM	1.0 mM	2.0 mM	0.5 mM	1.0 mM	2.0 mM	0.5 mM	1.0 mM	2.0 mM	0.5 mM	1.0 mM	2.0 mM
1	60.0	1.0	32.44	61.61	33.24	631.49	638.01	797.59	3054.00	1838.40	1217.67	30.03	41.97	39.93	48.30	33.10	38.40
2	40.0	3.0	26.40	24.44	53.56	808.99	844.81	613.58	974.55	1988.41	4104.08	31.69	43.71	43.63	44.00	38.30	31.50
3	60.0	3.0	56.87	62.08	61.58	685.23	668.95	760.14	2541.90	4683.43	3359.20	50.55	31.05	28.93	32.20	29.40	30.00
4	64.1	2.0	23.46	59.88	28.06	848.07	839.93	792.70	1434.93	2366.03	3809.23	41.41	46.77	49.11	31.50	31.70	34.10
5	50.0	0.6	32.43	31.30	32.10	652.66	662.43	743.85	1440.10	3907.51	4797.23	32.45	51.05	40.96	39.60	36.90	34.50
6	60.0	1.0	32.44	61.61	33.24	631.49	638.01	797.59	3054.00	1838.40	1217.67	30.03	41.97	39.93	48.30	33.10	38.40
7	60.0	3.0	56.87	62.08	61.58	685.23	668.95	760.14	2541.90	4683.43	3359.20	50.55	31.05	28.93	32.20	29.40	30.00
8	40.0	1.0	41.80	44.97	43.51	756.88	1017.42	815.50	1165.94	2671.22	1512.52	43.68	56.87	46.31	30.10	20.90	29.00
9	50.0	2.0	33.55	28.87	24.17	696.63	771.54	704.77	1734.95	1491.83	1631.49	48.57	41.57	50.55	26.20	29.80	29.10
10	50.0	0.6	32.43	31.30	32.10	652.66	662.43	743.85	1440.10	3907.51	4797.23	32.45	51.05	40.96	39.60	36.90	34.50
11	60.0	3.0	56.87	62.08	61.58	685.23	668.95	760.14	2541.90	4683.43	3359.20	50.55	31.05	28.93	32.20	29.40	30.00
12	40.0	3.0	26.40	24.44	53.56	808.99	844.81	613.58	974.55	1988.41	4104.08	31.69	43.71	43.63	44.00	38.30	31.50
13	50.0	3.4	56.06	51.49	54.40	621.72	603.81	600.55	5966.27	4093.73	4445.48	38.48	50.24	38.04	36.00	36.60	40.80
14	50.0	3.4	56.06	51.49	54.40	621.72	603.81	600.55	5966.27	4093.73	4445.48	38.48	50.24	38.04	36.00	36.60	40.80
15	40.0	1.0	41.80	44.97	43.51	756.88	1017.42	815.50	1165.94	2671.22	1512.52	43.68	56.87	46.31	30.10	20.90	29.00
16	60.0	1.0	32.44	61.61	33.24	631.49	638.01	797.59	3054.00	1838.40	1217.67	30.03	41.97	39.93	48.30	33.10	38.40
17	50.0	0.6	32.43	31.30	32.10	652.66	662.43	743.85	1440.10	3907.51	4797.23	32.45	51.05	40.96	39.60	36.90	34.50
18	35.9	2.0	34.50	38.08	35.76	813.87	740.60	934.38	1352.16	2086.70	3385.06	42.66	49.29	42.34	32.00	32.90	35.00
19	64.1	2.0	23.46	59.88	28.06	848.07	839.93	792.70	1434.93	2366.03	3809.23	41.41	46.77	49.11	31.50	31.70	34.10
20	50.0	2.0	33.55	28.87	24.17	696.63	771.54	704.77	1734.95	1491.83	1631.49	48.57	41.57	50.55	26.20	29.80	29.10
21	35.9	2.0	34.50	38.08	35.76	813.87	740.60	934.38	1352.16	2086.70	3385.06	42.66	49.29	42.34	32.00	32.90	35.00
22	50.0	2.0	33.55	28.87	24.17	696.63	771.54	704.77	1734.95	1491.83	1631.49	48.57	41.57	50.55	26.20	29.80	29.10
23	50.0	2.0	33.55	28.87	24.17	696.63	771.54	704.77	1734.95	1491.83	1631.49	48.57	41.57	50.55	26.20	29.80	29.10
24	35.9	2.0	34.50	38.08	35.76	813.87	740.60	934.38	1352.16	2086.70	3385.06	42.66	49.29	42.34	32.00	32.90	35.00
25	40.0	3.0	26.40	24.44	53.56	808.99	844.81	613.58	974.55	1988.41	4104.08	31.69	43.71	43.63	44.00	38.30	31.50
26	64.1	2.0	23.46	59.88	28.06	848.07	839.93	792.70	1434.93	2366.03	3809.23	41.41	46.77	49.11	31.50	31.70	34.10
27	50.0	2.0	33.55	28.87	24.17	696.63	771.54	704.77	1734.95	1491.83	1631.49	48.57	41.57	50.55	26.20	29.80	29.10
28	50.0	3.4	56.06	51.49	54.40	621.72	603.81	600.55	5966.27	4093.73	4445.48	38.48	50.24	38.04	36.00	36.60	40.80
29	40.0	1.0	41.80	44.97	43.51	756.88	1017.42	815.50	1165.94	2671.22	1512.52	43.68	56.87	46.31	30.10	20.90	29.00

**Table 4:** ANOVA (analysis of variance) and estimated coefficients of the fitted model representing lack of fit, sum of squares, and R^2^ of RSM model.

**Response**	**Source**	**Sum of Squares**	**Mean Squares**	**DF**	***F*-Test**	***p*-Value**
**Antioxidant**	**Model**	15.98	3.2	5	13.52	<0.0001
	**Lack of Fit**	5.44	1.81	3		
	**Residual**	5.44	0.2365	23		
	**R²**	0.8997				
**TPC**	**Model**	59.94	8.56	7	631.91	<0.0001
	**Lack of Fit**	0.2846	0.2846	1		
	**Residual**	0.2846	0.0136	21		
	**R²**	0.9953				
**TFC**	**Model**	4866.9	695.27	7	110.18	<0.0001
	**Lack of Fit**	132.5	132.5	1		
	**Residual**	132.5	6.31	21		
	**R²**	0.9735				
**Anti-tyrosinase**	**Model**	9.05	1.81	5	458.09	<0.0001
	**Lack of Fit**	0.0909	0.0303	3		
	**Residual**	0.0909	0.004	23		
	**R²**	0.9901				
**Extraction Yield**	**Model**	8.87	1.77	5	60.44	<0.0001
	**Lack of Fit**	0.6751	0.225	3		
	**Residual**	0.6751	0.0294	23		
	**R²**	0.9873				
**Sodium Silicate 0.5 mM**						
**Response**	**Source**	**Sum of Squares**	**Mean Squares**	**DF**	***F*-Test**	***p*-Value**
**Antioxidant**	**Model**	15.98	3.2	5	13.52	<0.0001
	**Lack of Fit**	5.44	1.81	3		
	**Residual**	5.44	0.2365	23		
	**R²**	0.8997				
**TPC**	**Model**	59.94	8.56	7	631.91	<0.0001
	**Lack of Fit**	0.2846	0.2846	1		
	**Residual**	0.2846	0.0136	21		
	**R²**	0.9953				
**TFC**	**Model**	4866.9	695.27	7	110.18	<0.0001
	**Lack of Fit**	132.5	132.5	1		
	**Residual**	132.5	6.31	21		
	**R²**	0.9735				
**Anti-tyrosinase**	**Model**	9.05	1.81	5	458.09	<0.0001
	**Lack of Fit**	0.0909	0.0303	3		
	**Residual**	0.0909	0.004	23		
	**R²**	0.9901				
**Extraction Yield**	**Model**	8.87	1.77	5	60.44	<0.0001
	**Lack of Fit**	0.6751	0.225	3		
	**Residual**	0.6751	0.0294	23		
	**R²**	0.9873				
**Sodium Silicate 1.0 mM**						
**Response**	**Source**	**Sum of Squares**	**Mean Squares**	**DF**	***F*-Test**	***p*-Value**
**Antioxidant**	**Model**	26.25	5.25	5	16.34	<0.0001
	**Lack of Fit**	7.38	2.46	3		
	**Residual**	7.38	0.32	23		
	**R²**	0.99				
**TPC**	**Model**	118.74	16.96	7	30.51	<0.0001
	**Lack of Fit**	68.78	34.39	2		
	**Residual**	68.78	3.13	22		
	**R²**	0.95				
**TFC**	**Model**	2794.58	558.91	5	61.09	<0.0001
	**Lack of Fit**	210.41	70.13	3		
	**Residual**	210.41	9.14	23		
	**R²**	0.96				
**Anti-tyrosinase**	**Model**	6.47	0.92	7	14.52	<0.0001
	**Lack of Fit**	1.33	1.33	1		
	**Residual**	1.32	0.06	21		
	**R²**	0.93				
**Extraction Yield**	**Model**	4.08	0.81	5	11.19	<0.0001
	**Lack of Fit**	1.68	0.56	3		
	**Residual**	1.68	0.07	23		
	**R²**	0.89				
**Sodium Silicate 2.0 mM**						
**Response**	**Source**	**Sum of Squares**	**Mean Squares**	**DF**	***F*-Test**	***p*-Value**
**Antioxidant**	**Model**	24.67	4.93	5	27.81003	<0.0001
	**Lack of Fit**	4.08	1.36	3		
	**Residual**	4.08	0.17	23		
	**R²**	0.87				
**TPC**	**Model**	73.44	14.68	5	24.40022	<0.0001
	**Lack of Fit**	13.84	4.61	3		
	**Residual**	13.84	0.60	23		
	**R²**	0.85				
**TFC**	**Model**	3114.30	444.90	7	6.914045	<0.0001
	**Lack of Fit**	1351.29	1351.29	1		
	**Residual**	1351.29	64.34	21		
	**R²**	0.81				
**Anti-tyrosinase**	**Model**	6.80	0.97	7	57.31987	<0.0001
	**Lack of Fit**	0.35	0.35	1		
	**Residual**	0.35	0.01	21		
	**R²**	0.95				
**Extraction Yield**	**Model**	2.60	0.37	7	11.49911	<0.0001
	**Lack of Fit**	0.67	0.67	1		
	**Residual**	0.67	0.03	21		
	**R²**	0.8132				

## Data Availability

Data are contained within the article and supplementary materials.

## Supplementary Materials

The following supporting information can be downloaded at: https://www.mdpi.com/article/10.3390/antiox13010054/s1, Figure S1: Calibration curve of 12 different standards used for quantification analysis; Figure S2: A–B represents TIC of control, C–D represents TIC of Aerial treatment and E-F represents Media data. Figure S1 displayed calibration curve of each ginsenosides separately and Figure S2, represents a combined TIC of Control, aerial and media treatment.
