# Supplementary material for: Impact of Sodium Silicate Supplemented, IR-Treated Panax Ginseng on Extraction Optimization for Enhanced Anti-Tyrosinase and Antioxidant Activity: A Response Surface Methodology (RSM) Approach
Source: Antioxidants (Basel). 2023 Dec 28;13(1):54. doi: 10.3390/antiox13010054 (PMC10812770; doi:10.3390/antiox13010054)
Supplement: Supplementary file 1 [file antioxidants-13-00054-s001.zip › antioxidants-2743050-supplementary.pdf]

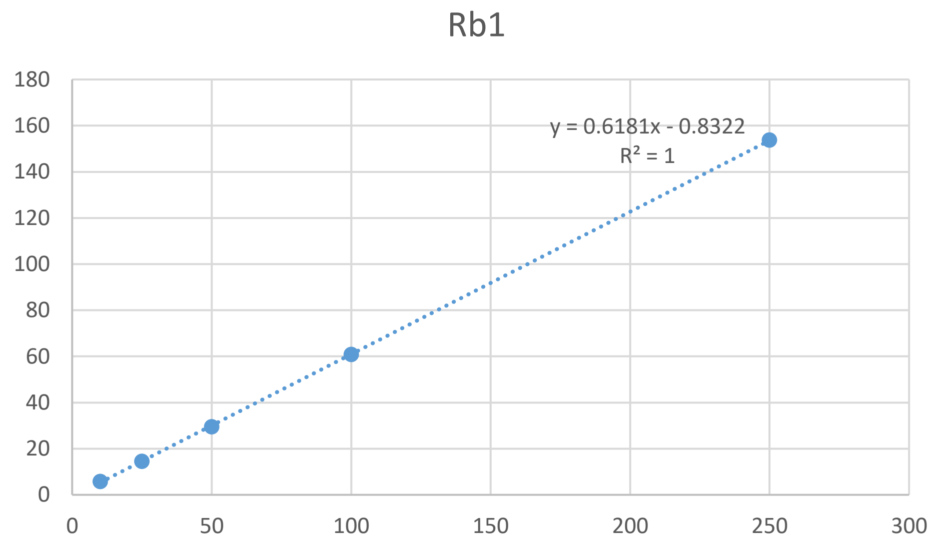
Rb1
y = 0.6181x - 0.8322
R² = 1
0 20 40 60 80 100 120 140 160 180
0 50 100 150 200 250 300

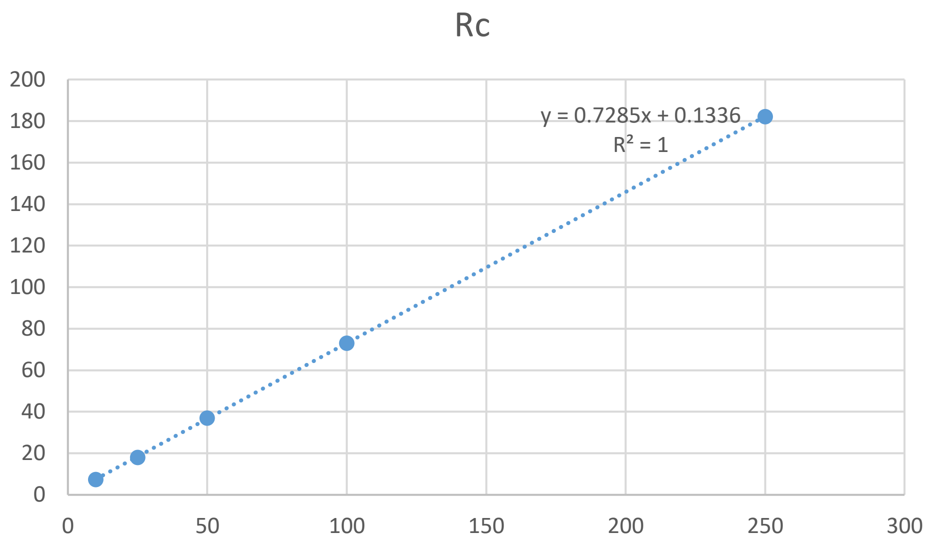
Rc
y = 0.7285x + 0.1336
R² = 1
0 20 40 60 80 100 120 140 160 180 200
0 50 100 150 200 250 300

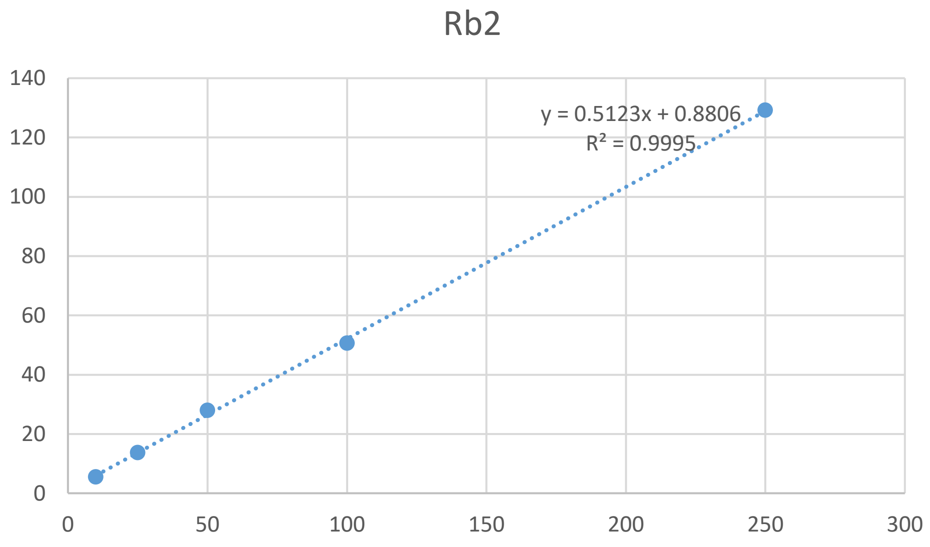
Rb2
y = 0.5123x + 0.8806
R² = 0.9995
0 20 40 60 80 100 120 140
0 50 100 150 200 250 300

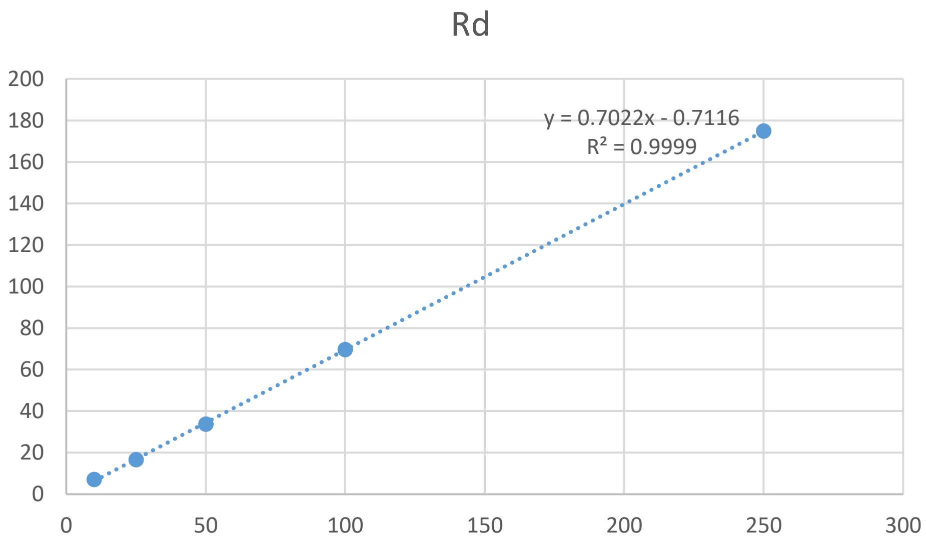
Rd
y = 0.7022x - 0.7116
R² = 0.9999
0 20 40 60 80 100 120 140 160 180 200
0 50 100 150 200 250 300

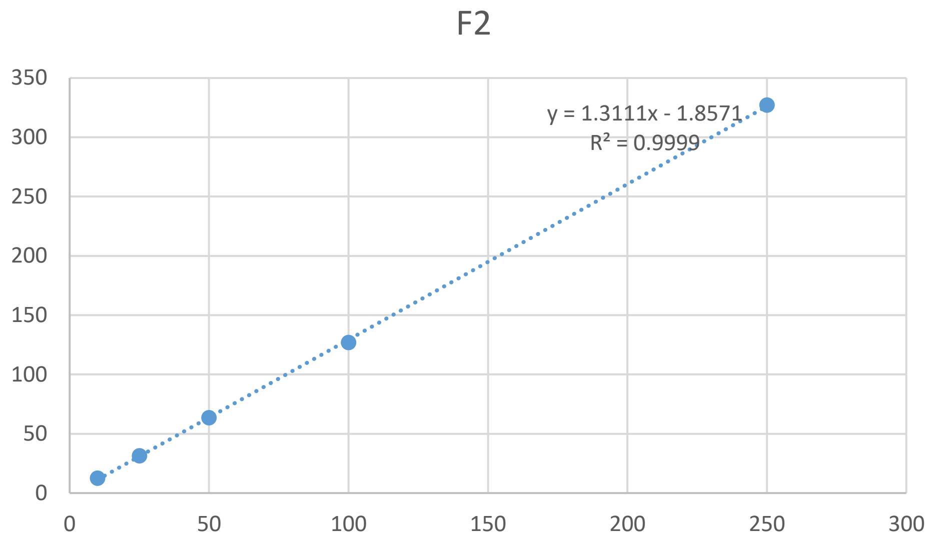
F2
y = 1.3111x - 1.8571
R² = 0.9999
0
50
100
150
200
250
300
350
0
50
100
150
200
250
300

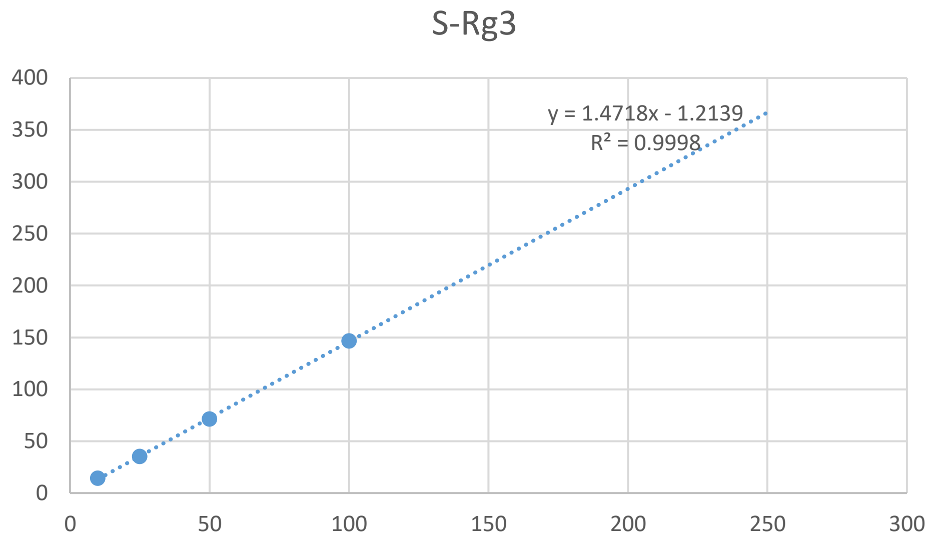
S-Rg3
y = 1.4718x - 1.2139
R² = 0.9998
0
50
100
150
200
250
300
350
400
0
50
100
150
200
250
300

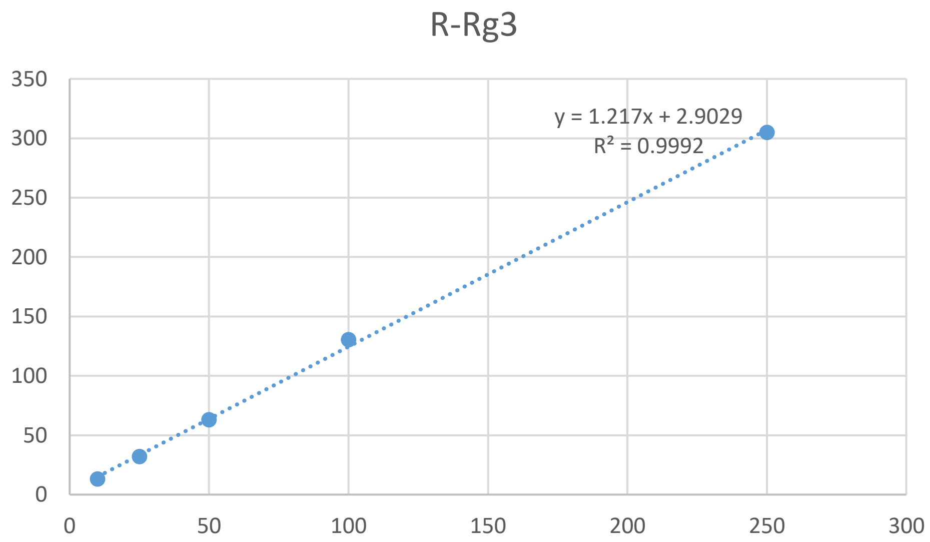
R-Rg3
y = 1.217x + 2.9029
R² = 0.9992
0
50
100
150
200
250
300
350
0
50
100
150
200
250
300

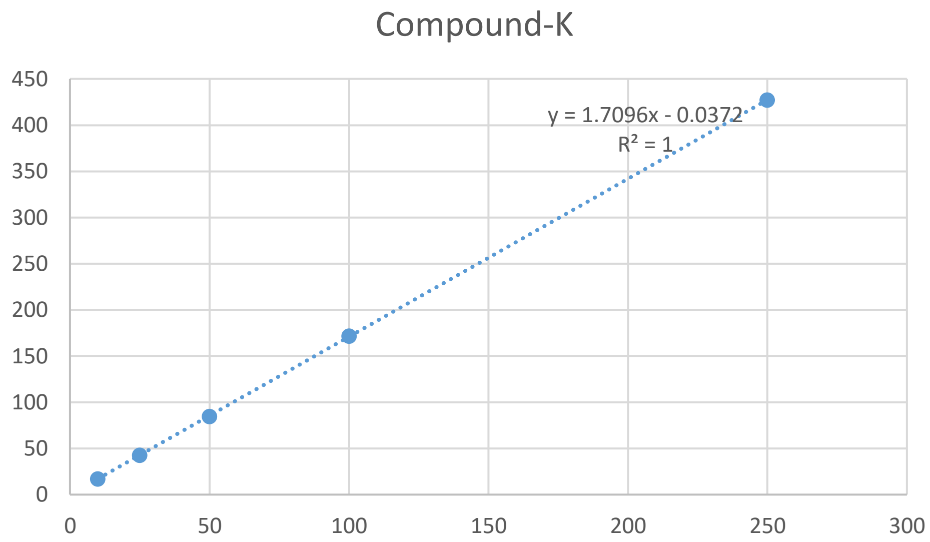
Compound-K
y = 1.7096x - 0.0372
R² = 1
0
50
100
150
200
250
300
350
400
450
0
50
100
150
200
250
300

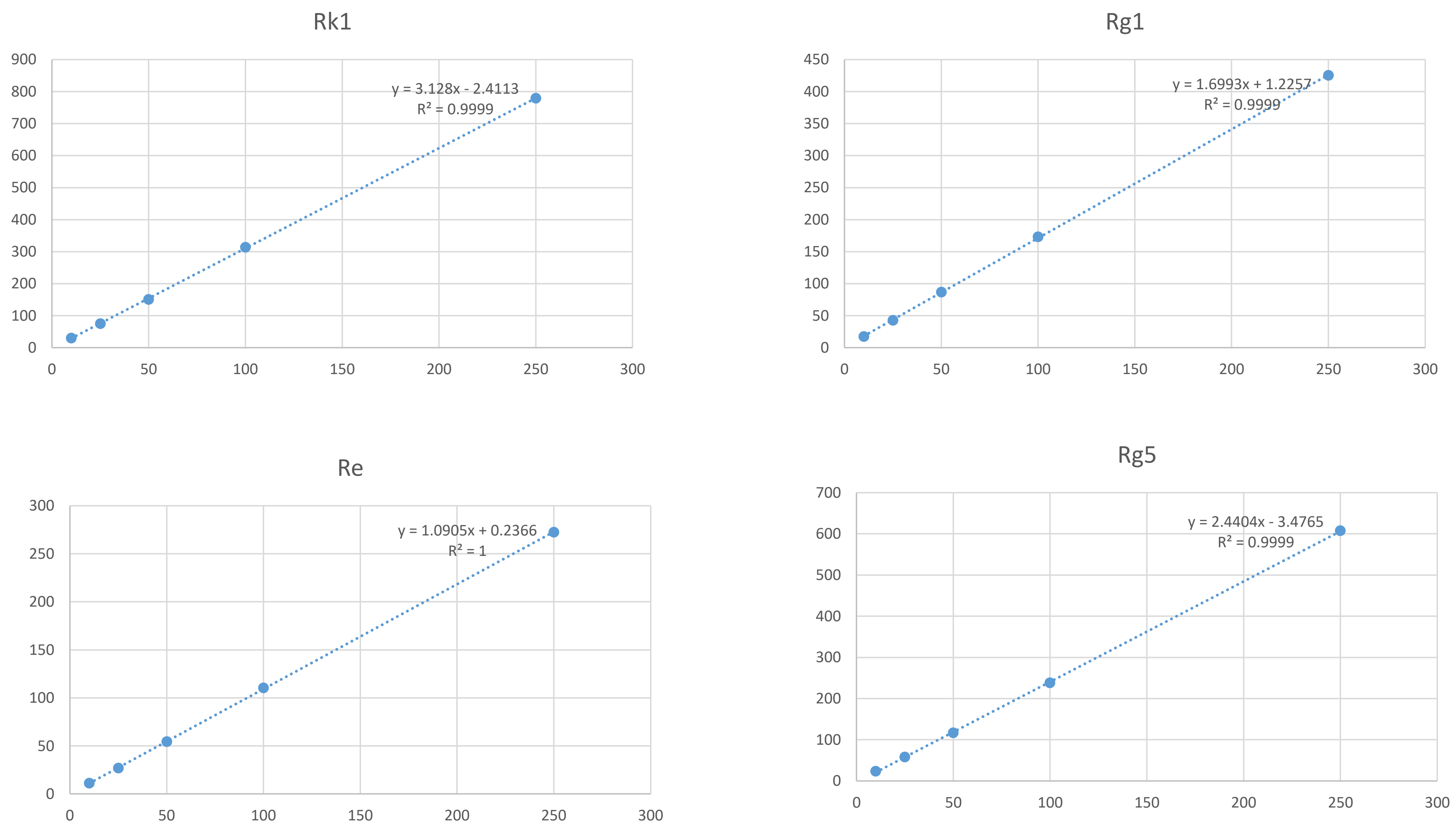

Supplementary Figure S1. Calibration curve of 12 different standards used for quantification analysis

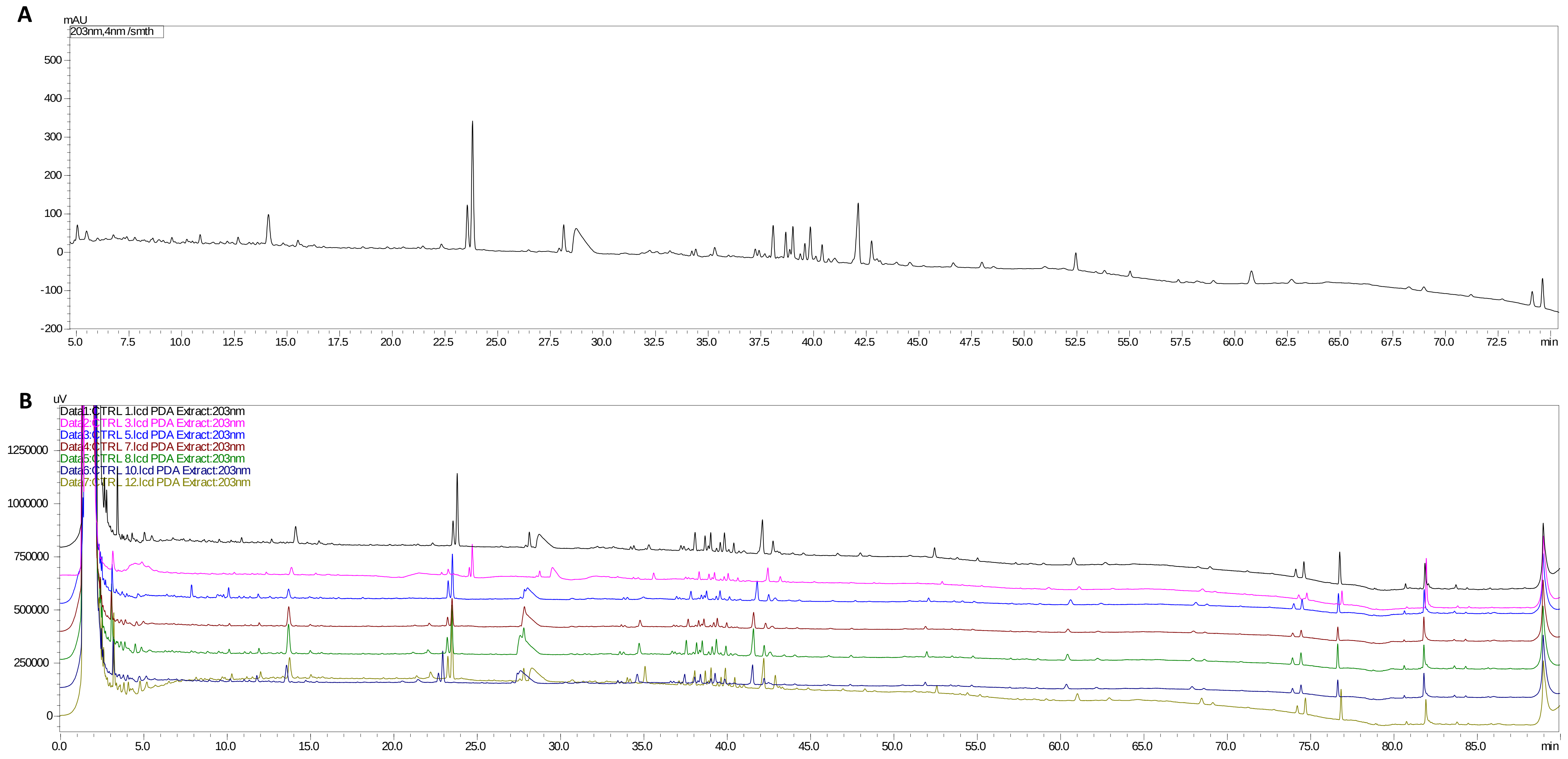
A
mAU
203nm,4nm /smth
500
400
300
200
100
0
-100
-200
5.0
7.5
10.0
12.5
15.0
17.5
20.0
22.5
25.0
27.5
30.0
32.5
35.0
37.5
40.0
42.5
45.0
47.5
50.0
52.5
55.0
57.5
60.0
62.5
65.0
67.5
70.0
72.5
min
B
uV
Data1:CTRL 1.lcd PDA Extract:203nm
Data2:CTRL 3.lcd PDA Extract:203nm
Data3:CTRL 5.lcd PDA Extract:203nm
Data4:CTRL 7.lcd PDA Extract:203nm
Data5:CTRL 8.lcd PDA Extract:203nm
Data6:CTRL 10.lcd PDA Extract:203nm
Data7:CTRL 12.lcd PDA Extract:203nm
1250000
1000000
750000
500000
250000
0
0.0
5.0
10.0
15.0
20.0
25.0
30.0
35.0
40.0
45.0
50.0
55.0
60.0
65.0
70.0
75.0
80.0
85.0
min

C

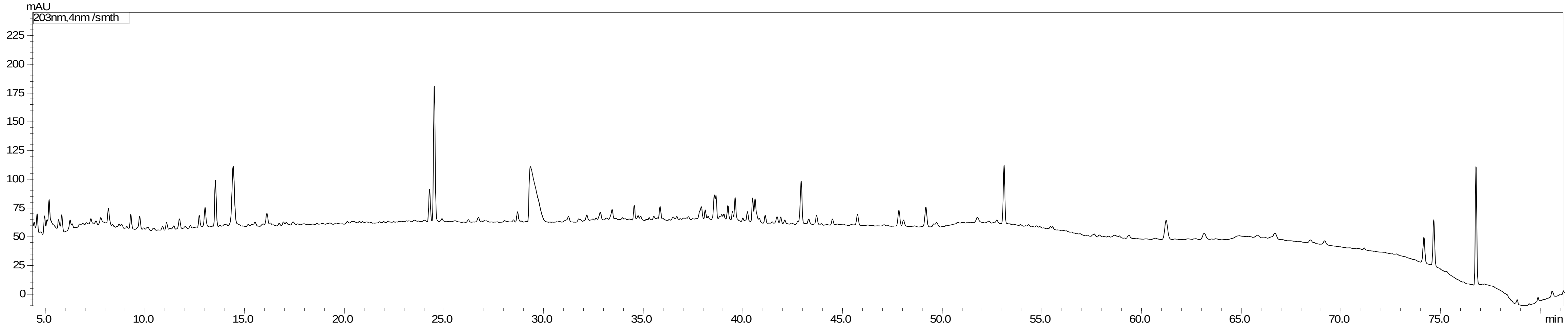

mAU
203nm,4nm /smth
225
200
175
150
125
100
75
50
25
0
5.0
10.0
15.0
20.0
25.0
30.0
35.0
40.0
45.0
50.0
55.0
60.0
65.0
70.0
75.0
min

D

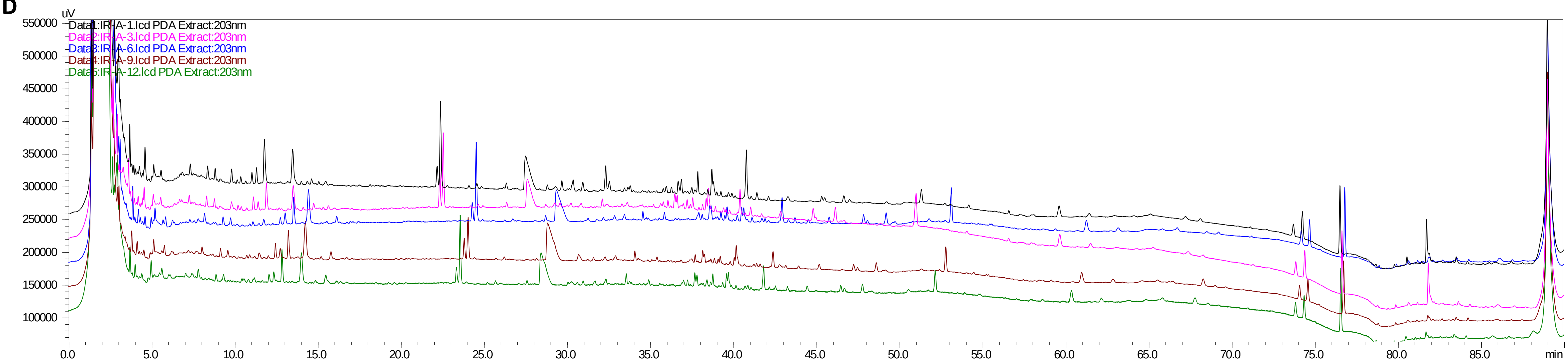

uV
Data1:IR-A-1.lcd PDA Extract:203nm
Data2:IR-A-3.lcd PDA Extract:203nm
Data3:IR-A-6.lcd PDA Extract:203nm
Data4:IR-A-9.lcd PDA Extract:203nm
Data5:IR-A-12.lcd PDA Extract:203nm
550000
500000
450000
400000
350000
300000
250000
200000
150000
100000
0.0
5.0
10.0
15.0
20.0
25.0
30.0
35.0
40.0
45.0
50.0
55.0
60.0
65.0
70.0
75.0
80.0
85.0
min

E

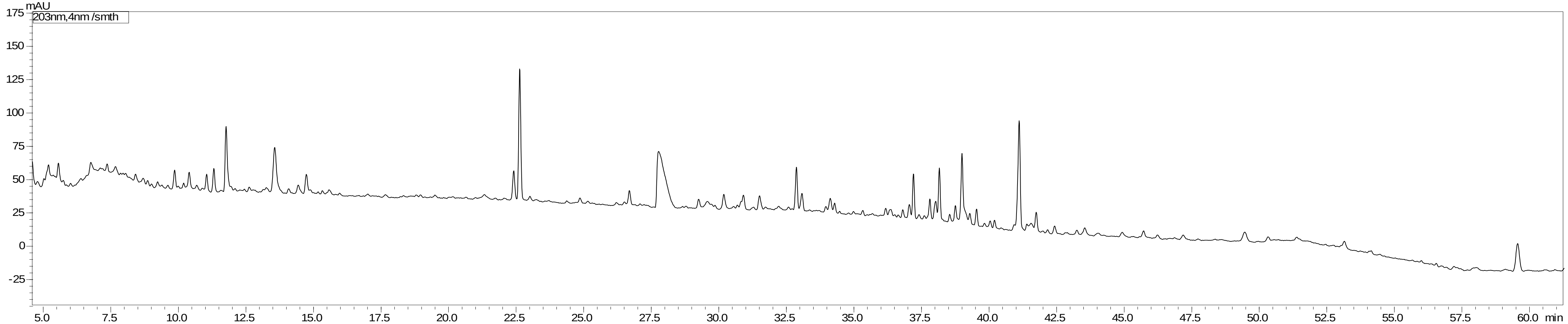

F

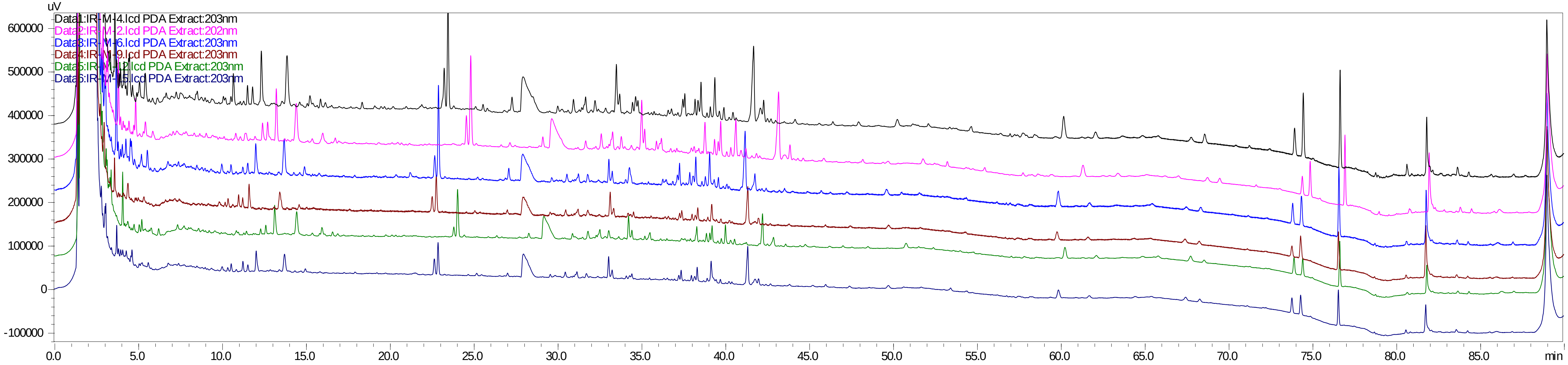

Supplementary Figure S2. A-B represents TIC of control, C-D represents TIC of Aerial treatment and E-F represents Media data
